# Independent and interactive associations of heart rate and obesity with type 2 diabetes mellites: A population‐based study

**DOI:** 10.1111/1753-0407.13529

**Published:** 2024-04-10

**Authors:** Tianxin Zhu, Qingyu Chen, Hongxing Chen, Lili You, Dan Liu, Xiaoyun Zhang, Feng Li, Hongshi Wu, Juying Tang, Diaozhu Lin, Kan Sun, Li Yan, Meng Ren

**Affiliations:** ^1^ Department of Endocrinology, Sun Yat‐sen Memorial Hospital Sun Yat‐sen University Guangzhou China; ^2^ Health Examination Center, Sun Yat‐sen Memorial Hospital Sun Yat‐sen University Guangzhou China

**Keywords:** heart rate, interactive effects, obesity, type 2 diabetes mellites

## Abstract

**Background:**

Although obesity and heart rate (HR) were closely related to the prevalence and development of type 2 diabetes mllitus (T2DM), few studies have shown a co‐association effect of them on T2DM. We aimed at assessing the interactive effects of HR and obesity with prevalence of T2DM in Chinese population, providing the exact cutpoint of the risk threshold for blood glucose with high HR.

**Materials and Methods:**

In the Risk Evaluation of cAncers in Chinese diabeTic Individuals: a lONgitudinal study (REACTION) cohorts (*N* = 8398), the relationship between HR and T2DM was explored by linear regression, logistic regression, and restricted cubic spline, and odds ratios (ORs) and 95% confidence intervals (CIs) were calculated. Interaction terms between HR and body mass index (BMI) and HR and waist circumference (WC) were introduced into the logistic regression model.

**Results:**

In those with HR > 88.0 beats/min, fasting plasma glucose and oral glucose tolerance tests were significantly correlated with HR, and the prevalence of T2DM was highly correlated with HR (all *p* < .05). There were interactive associations of HR and obesity in patients with T2DM with HR < 74 beats/min.

**Conclusion:**

High HR was in interaction with obesity, associating with prevalence of T2DM. The newly subdivided risk threshold for HR with T2DM might be HR > 88 beats/minute.

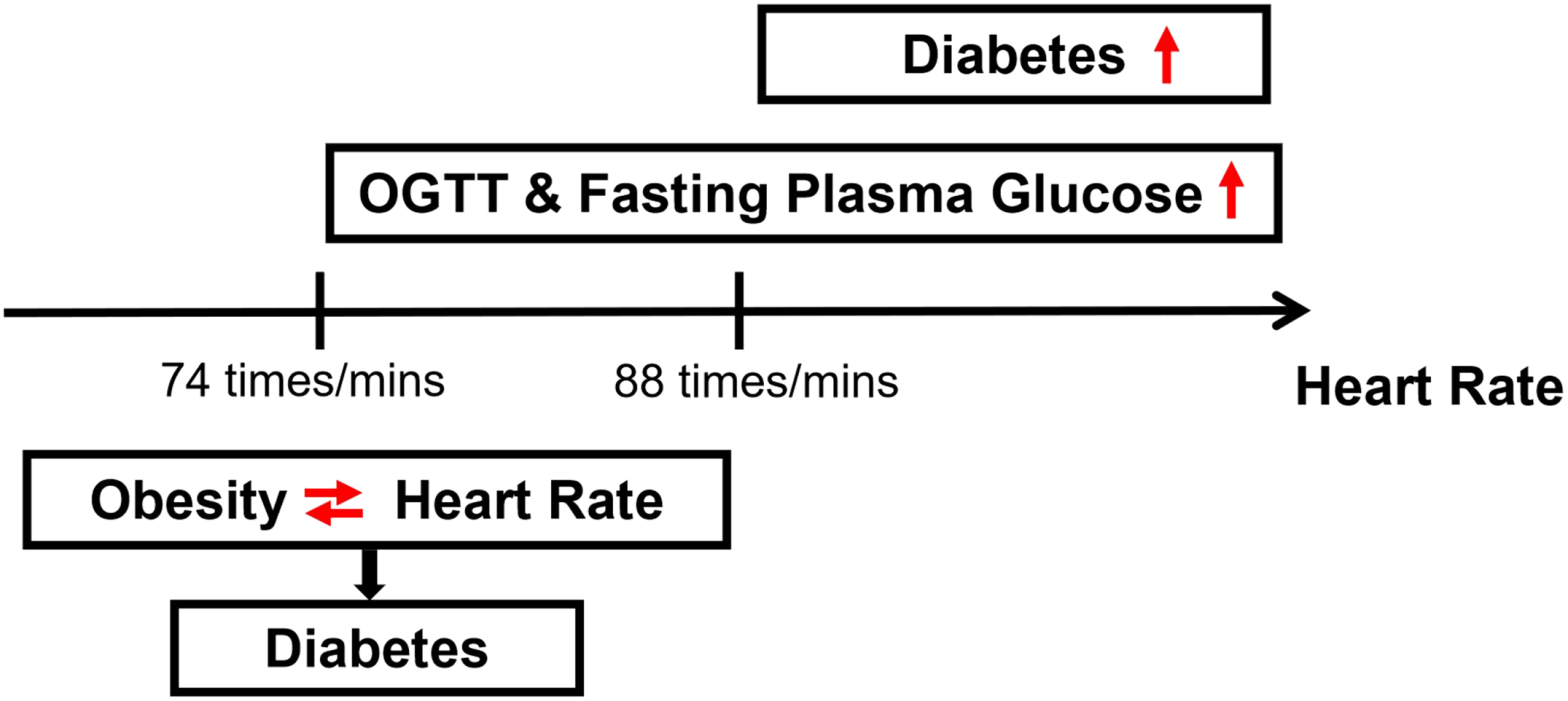

## Graphical abstract

Highlights

- With the increase of heart rate (HR) level, high levels HR was associated with increasing risk of type 2 diabetes mellitus (T2DM).
- HR was in interaction with obesity, associating with incidence of T2DM in diabetic patients with HR < 74 beats/min.
- HR could be used as an independent assessment factor for diabetic patients with HR > 88 beats/min.

## 1 INTRODUCTION

Diabetes mellitus (DM) is a group of metabolic diseases characterized by hyperglycemia. According to the latest report of the International Diabetes Federation, the crude prevalence of diabetes in the world was 9.3% in 2019, and it was expected to be 10.2% and 10.9% in 2030 and 2045. China, having the largest number of patients with diabetes in 2019 with a total number of 116 million, will continue to take the top position with 140 million and 147 million of patients with diabetes in 2030 and 2045, respectively.^1^ As a kind of lifelong disease, DM will not only cause disability, death, and serious prognosis but also lead to heavy economic burden on society and families. Accordingly, increasing attention has been paid to the early prediction and diagnosis of DM.

Obesity is an increasingly prevalent metabolic disease that has reached alarming levels, and it was estimated that the number of obese people has increased from 105 million in 1975 to 641 million in 2014 all over the world.^2^ Obesity is currently the strongest and most widely studied risk factor in type 2 DM (T2DM).^3^, ^4^ According to the study of Chinese Guidelines for the Prevention and Treatment of Type 2 Diabetes (2017 edition), the prevalence of T2DM in obese people doubled. Many reports^3^, ^4^ show that weight loss in overweight patients with glucose intolerance has a better effect on preventing or managing T2DM.

Heart rate (HR) was used as a routine clinical measure and potential prognostic marker as it was associated with various chronic diseases^5^, ^6^, ^7^ and all‐cause mortality.^8^ The HR might be directly related to the development of T2DM. A few prospective cohort studies have found that accelerated HR might predict the development of T2DM in different populations.^9^, ^10^ The rise of HR reflected the disorder of the autonomic nerve system,^11^, ^12^ which led to the excitation of sympathetic nerves,^13^ which could result in obesity, decreased insulin sensitivity, inflammation, and the increasing risk of T2DM.^14^, ^15^ Nevertheless, although the aforementioned studies have shown that fast HR could be associated with the prevalence of T2DM, there was no clear risk threshold for HR to be a common accompaniment to T2DM.

As mentioned, the relationship between HR, obesity, and T2DM^13^ has attracted increased attention. With the deepening of disease research, researchers are no longer limited to focus on the influence of the single factor on disease, and the interaction of various factors in disease has gradually been valued by people.^16^ At present, studies have shown that there is an important correlation between HR and obesity in diseases^17^, ^18^; we therefore hypothesized that the single and interactive effects of HR and obesity are crucial to the occurrence of T2DM. However, there are few studies^4^ on the interactive effects between HR and obesity on T2DM at home and abroad, and the large sample and high‐quality studies are still needed to further demonstrate. Consequently, we analyzed data from a Chinese population to explore the independent and interactive associations of HR and obesity with T2DM and provide the possible risk threshold for HR with T2DM.

## 2 SUBJECTS, MATERIALS, AND METHODS

### 2.1 Study population and design

**FIGURE 1:**
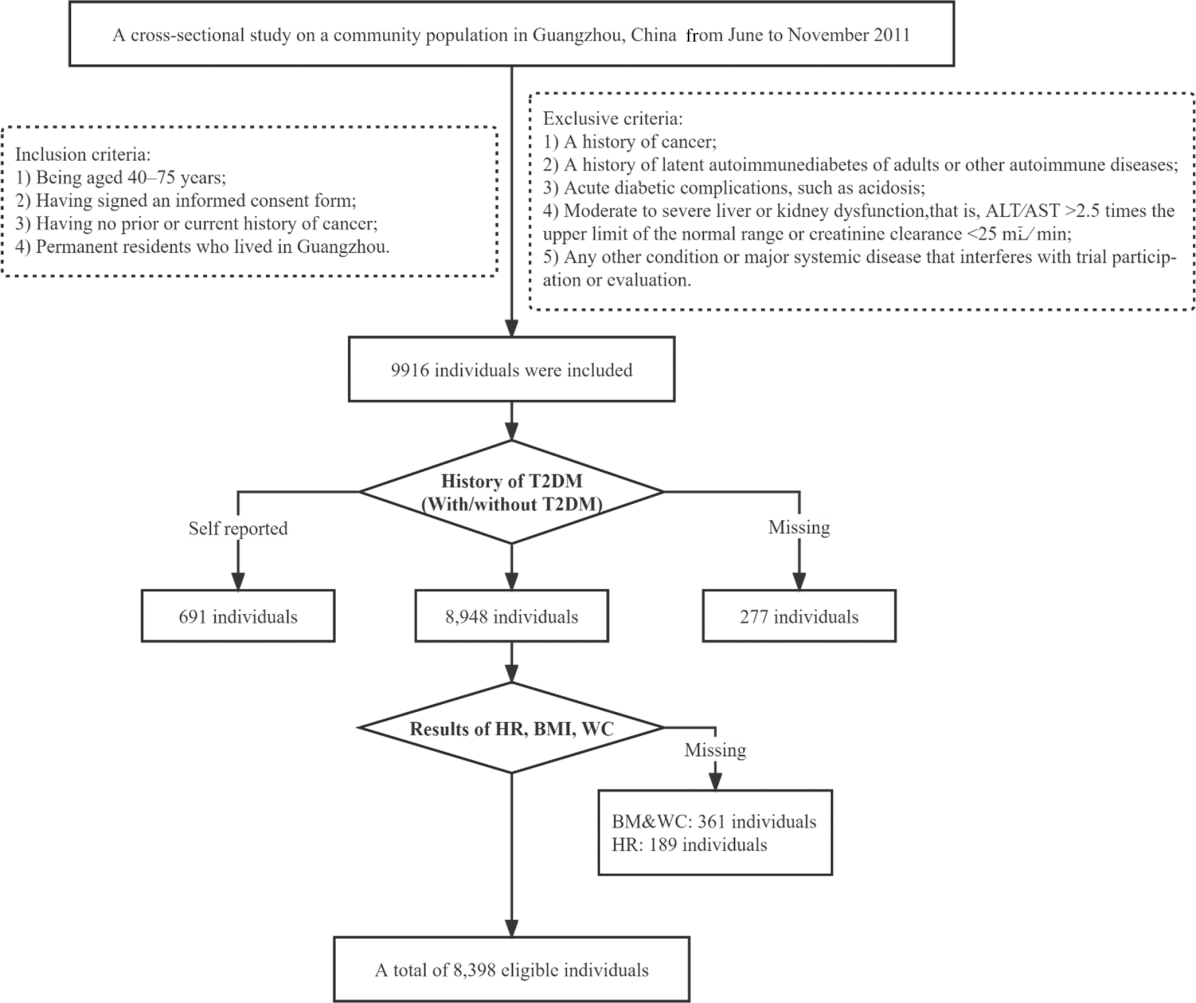
Screening flow chart of participants. ALT, alanine transaminase; AST, aspartate transaminase; BMI, body mass index; HR, heart rate; T2DM, type 2 diabetes mellitus; WC, waist circumference.

We conducted a cross‐sectional study on a community population in Guangzhou, China from June to November 2011, and the research population came from the Risk Evaluation of cAncers in Chinese diabeTic Individuals: A lONgitudinal (REACTION) study, which was a multicenter prospective observational study to assess chronic diseases in Chinese.^19^, ^20^ The study population, design, and protocol were similar to the previous studies.^21^ A total of 10 104 residents aged 40 and above participated through examination or the home visit, and 9916 individuals signed the consent form and agreed to participate in the survey; the participation rate was 98.1%. Due to our study focus on T2DM, obesity, and HR, subjects with inappropriate T2DM information to eliminate the influence of medication (self‐reported T2DM, *n* = 691; missing T2DM test results, *n* = 189; missing history of newly diagnosed T2DM, *n* = 88) and missing other relevant data (body mass index [BMI], *n* = 273; waist circumference [WC], *n* = 203; and HR, *n* = 189) were excluded from the analysis. Consequently, there were 8398 eligible individuals in the final data analysis. The detailed flow chart for screening is shown in Figure 1.

The study has been approved by the Ethics Committee of Sun Yat‐sen Memorial Hospital, Sun Yat‐sen University (approval number 2014‐033), and strictly abides by the principles of Helsinki declaration. All participants had written informed consent for data collection and publication.

### 2.2 Clinical and biochemical measurements

We used standardized questionnaires to collect information about participants' demographic characteristics (marital status, employment, and educational level), lifestyle (smoking and drinking), medical history, and family history. The educational level was divided into primary school and below, junior middle school, technical secondary school or senior high school, undergraduate and above. Smoking or drinking habits are classified as “never,” “current” (frequent smoking or drinking in the past 6 months), or “ever” (quitting smoking or drinking for more than 6 months).^22^

All participants completed physical examination with standard protocols under the assistance of trained staff. Blood pressure and HR were measured three times by the same observer with an electronic sphygmomanometer (OMRON, Omron Company, Dalian, China). The mean of three blood pressure measurements and the mean of three HR measurements were used for analysis. When measuring these indicators, we required participants to maintain sufficient sleep the night before and prohibit exercise, smoking, and caffeine intake for the first 30 min of measurement. Participants were required to rest quietly for 5 min and were measured in their seated position. The cuff was placed at the level of the heart during the measurement, and all participants were measured using the same cuff. The height and weight are accurate to 0.1 cm and 0.1 kg. BMI was calculated as the weight divided by the square of height (kg/m^2^). The waistline, which also known as WC, was measured at the umbilical level with measuring tape when participants were in the standing position and at the end of gentle exhalation. Hip circumference (HC) was measured at the most convex part of the hip bone and pubic symphysis, with the legs tightly together and the arms resting on both sides.

After the participants experienced fasting at night for at least 10 h, venous blood samples were collected for laboratory tests, including fasting plasma glucose (FPG), triglycerides, total cholesterol (CHOL), high‐density lipoprotein cholesterol (HDL), low‐density lipoprotein cholesterol (LDL), creatinine, γ‐glutamyltransferase (GGT), aspartate aminotransferase (AST), and alanine aminotransferase (ALT) (Beckman CX‐7 automatic biochemical analyzer, California, USA). Hemoglobin A1c (HbA1c) was evaluated by high performance liquid chromatography (Bio‐Rad, Hercules, CA).

### 2.3 Definition of diabetes mellitus and obesity

The diagnosis of T2DM was in accordance with the diagnostic criteria published by the Chinese Diabetes Society in 2020. This diagnostic standard requires that the tested participants have typical diabetes symptoms, combined with random blood glucose ≥11.1 mmol/L (≥200 mg/dL), or FPG ≥7.0 mmol/L (≥126 mg/dL), or oral glucose tolerance test (OGTT) 2 h ≥ 11.1 mmol/L (≥200 mg/dL), or HbA1c ≥ 6.5%. If the tested participants have no typical symptoms, the test results were rechecked on another day.

Obesity was defined as BMI ≥ 28, and overweight was defined as BMI ≥ 24 kg/m^2^ and < 28 kg/m^2^. The definition of central obesity for men was WC > 85 cm and for women WC > 80 cm.^23^

### 2.4 Statistical analysis

The continuous variables with normal distribution were expressed as mean ± SD, and *t* test was used to analyze the differences between groups. The continuous variables of nonnormal distribution data were expressed as median and interquartile range, and Kruskal–Wallis test was used to compare the differences between groups. Categorical variables are expressed as numbers (proportions). *χ* ^2^ test was used to compare the classification variables. Restricted cubic spline models were applied to examine the possible nonlinear association between HR and T2DM in general population and gender‐specific participants, and the relevant covariates were adjusted: sex, age, marital status, DM family, educational level, CHOL, AST, HC, BMI, HDL, systolic blood pressure (SBP), WC, and GGT. We used unadjusted and multivariate linear regression to evaluate the association of HR with FPG, OGTT, and HbA1c. In addition, we applied unadjusted and multivariate logistic regression analysis to evaluate the effect of HR on T2DM (yes/no). Odds ratio (OR) and corresponding 95% confidence interval (95% CI) were calculated. To determine whether obesity interact with HR in prevalence of T2DM, product terms for BMI*HR or WC*HR were added to the regression model. The adjustment was then applied for relevant covariates (age, sex, marital status, T2DM family, educational level, CHOL, AST, HC, HDL, SBP, and GGT) in multivariate linear regression and multivariate logistic regression analysis. RStudio version 3.6.l was applied for all statistical analysis. All statistical tests were bilateral, and *p* < .05 was statistically significant.

## 3 RESULTS

### 3.1 Clinical characteristics of the study population

Table 1 shows the characteristics of the study population with or without T2DM. Compared with the participants without diabetes, the obesity index (such as weight, BMI, WC, HC) and HR level of patients with T2DM were significantly higher (all *p* < .001). In addition, the levels of SBP, diastolic blood pressure, CHOL, TG, LDL, AST, ALT, and GGT in the participants with T2DM were higher than those in the participants without diabetes (all *p* < .001). The HDL in the patients with T2DM was lower than that in the participants without diabetes (*p* < .001).

**TABLE 1:** Characteristics of study population with or without T2DM.

Variables	Nondiabetes *N* = 7089	T2DM *N* = 1309	*p*_difference_
Male, *n* (%)	1974 (27.8%)	394 (30.1%)	.103
Age, mean (SD)	54.6 (6.79)	57.3 (7.29)	<.001
Elementary school and below, *n* (%)	713 (10.3%)	210 (16.4%)	<.001
Smoking, *n* (%)	694 (9.93%)	141 (11.0%)	.389
Drinking, *n* (%)	223 (3.20%)	57 (4.43%)	.046
History of T2DM family, *n* (%)	1067 (15.3%)	271 (21.1%)	<.001
Marriage, *n* (%)	6367 (90.4%)	1145 (87.9%)	.006
Height, mean (SD)	158 (7.27)	158 (7.16)	.120
Weight, mean (SD)	58.2 (8.69)	61.0 (8.68)	<.001
BMI, mean (SD)	23.3 (2.86)	24.6 (2.96)	<.001
WC, mean (SD)	80.5 (8.54)	84.9 (8.81)	<.001
HC, mean (SD)	93.4 (6.14)	94.9 (6.40)	<.001
SBP, mean (SD)	124 (14.7)	131 (14.7)	<.001
DBP, mean (SD)	74.5 (9.23)	77.2 (9.16)	<.001
CHOL, mean (SD)	5.19 (1.11)	5.38 (1.17)	<.001
TG, median (IQR)	1.17 [0.87–1.62]	1.49 [1.07–1.98]	<.001
HDL, mean (SD)	1.33 (0.34)	1.24 (0.33)	<.001
LDL, mean (SD)	3.11 (0.88)	3.25 (0.95)	<.001
AST, median (IQR)	18.0 [15.0–21.0]	19.0 [15.0–23.0]	<.001
ALT, median (IQR)	12.0 [9.00–16.0]	13.0 [10.0–18.0]	<.001
GGT, median (IQR)	18.0 [14.0–25.0]	23.0 [17.0–31.5]	<.001
HR, mean (SD)	80.5 (10.3)	83.1 (10.7)	<.001

*Note*: Data were means ± SD or medians (interquartile ranges) for skewed variables or numbers (proportions) for categorical variables. *p* values were for the *t* test or *χ* ^2^ analyses between the groups.

Abbreviations: ALT, alanine aminotransferase; AST, aspartate aminotransferase; BMI, body mass index; CHOL, cholesterol; DBP, diastolic blood pressure; FPG, fasting plasma glucose; GGT, γ‐glutamyltransferase; HC, hip circumference; HDL, high‐density lipoprotein; HR, heart rate; IQR, interquartile range; LDL, low‐density lipoprotein; SBP, systolic blood pressure; TC, total cholesterol; TG, triglycerides; T2DM, type 2 diabetes mellites; WC, waist circumference.

### 3.2 The relationship between HR and prevalence of T2DM in different populations

**FIGURE 2:**
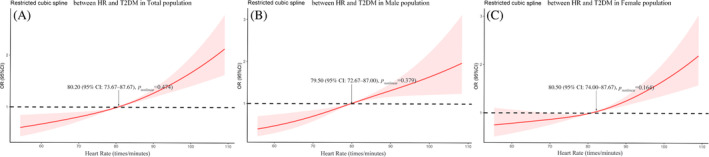
Heart rate (HR) and T2DM risk were analyzed by restricted cubic spline models. Odds ratios (OR) is represented by a red solid line, and 95% of confidence intervals (CIs) is represented by a red shaded part. (A) general population; (B) male population; (C) female population. T2DM, type 2 diabetes mellitus.

As shown in Figure 2, our study explored the relationship between HR and T2DM by restrictive cubic spline. The prevalence of T2DM showed an upward trend with the increase of HR level and there were differences in the odds ratio (OR) slope between HR and the prevalence of T2DM. The intersection points of the curve and the invalid line (OR = 1) were all around 80 beats/minute, and the 95% CI were given as 74–88 beats/min roughly. But there was no significant curvilinear correlation between HR and T2DM risk in the three populations (nonlinear *p* > .05).

### 3.3 The relationship between HR and indicators of T2DM in different HR subgroups

According to the results of restricted cubic spline models, we can explore the association between HR and T2DM prevalence by dividing HR into different subgroups shown in Table 2. We divided HR into three subgroups: HR < 74, 74 ≤ HR ≤ 88, and HR > 88 beats/min, and the relationship between HR and three indicators of DM (FPG, OGTT, and HbA1c) was explored by univariate and multivariate linear regression analysis. Linear regression analysis showed that HR was positively correlated with OGTT in different HR groups (all *p* < .05). HR was positively correlated with FPG in participants with 74 ≤ HR ≤ 88 and HR > 88 beats/min (all *p* < .05), but no significant positive correlation was found between HR and FPG in participants with HR < 74 beats/min. In addition, our results explored the relationship between HR and risk of T2DM by univariate and multivariate logistic regression analysis. Logistic regression analysis showed that there was a significant positive correlation between the increase of HR and the high risk of T2DM in the population with HR > 88 beats/min (OR = 1.028, 95% CI: 1.001–1.056, *p* = .041).

**TABLE 2:** Linear regression and logistic regression models of the relationship between HR and measures of glucose.

HR category	Linear regression molds for fold‐change per 1 unit increase in HR (95% CI)						Logistic regression molds for fold‐change per 1 unit increase in HR (95% CI)	
	FPG		OGTT		HbA1c		T2DM (yes vs no)	
	OR (95% CI)	*p*	OR (95% CI)	*p*	OR (95% CI)	*p*	OR (95% CI)	*p*
Unadjusted model								
HR < 74.0	1.003 (0.997–1.009)	.337	1.032 (1.013–1.052)	.001	1.001 (0.997–1.005)	.606	1.022 (0.992–1.055)	.161
74.0 ≤ HR ≤ 88.0	1.006 (1.001–1.011)	.010	1.023 (1.008–1.039)	.003	1.001 (0.998–1.004)	.488	1.014 (0.994–1.036)	.175
HR > 88.0	1.013 (1.008–1.019)	<.001	1.057 (1.038–1.076)	<.001	0.998 (0.995–1.002)	.369	1.034 (1.014–1.055)	<.001
Adjusted model^a^								
HR < 74.0	1.006 (0.999–1.012)	.083	1.042 (1.021–1.062)	<.001	1.002 (0.998–1.007)	.245	1.036 (0.996–1.079)	.086
74.0 ≤ HR ≤ 88.0	1.007 (1.002–1.012)	.003	1.027 (1.011–1.043)	.001	1.002 (0.999–1.006)	.143	1.023 (0.997–1.051)	.088
HR > 88.0	1.010 (1.004–1.016)	.001	1.035 (1.016–1.055)	<.001	0.999 (0.995–1.003)	.578	1.028 (1.001–1.056)	.041

Abbreviations: AST, aspartate aminotransferase; BMI, body mass index; CHOL, cholesterol; CI, confidence interval; FPG, fasting plasma glucose; GGT, γ‐glutamyltransferase; HbA1c, hemoglobin A1c; HC, hip circumference; HDL, high‐density lipoprotein; HR, heart rate; OGTT, oral glucose tolerance test; SBP, systolic blood pressure; T2DM, type 2 diabetes mellitus; WC, waist circumference.

^a^ Adjusted for: marriage + CHOL + educational level + AST + HC + BMI + HDL + SEX+SBP + WC + T2DM_family + age + GGT.

### 3.4 The interaction associated HR and obesity with the prevalence of T2DM

As shown in Table 3, we divided HR into three subgroups for analysis according to the stated criteria as well. In univariate analyses, greaterWC and BMI in different HR subgroups were associated with the prevalence of T2DM. When adjusted for relevant covariates, only in the participants with HR < 74 beats/min and participants with 74 ≤ HR ≤ 88 beats/min, greater WC and BMI remained positively associated with T2DM. The ORs of high BMI for increasing prevalence of T2DM was 1.17 (95% CI: 1.06–1.29, *p* = .002) for subjects with HR < 74 beats/min and 1.07 (95% CI: 1.01–1.14, *p* = .021) for participants with74 ≤ HR ≤ 88 beats/min, and the ORs of WC for risk of T2DM were 1.07 (95% CI: 1.03–1.11, *p* = .001) and 1.03 (95% CI: 1.01–1.06, *p* = .003), respectively.

**TABLE 3:** Associations between measures of obesity and T2DM in different HR groups.

HR category	Associated fold‐change (95% confidence interval) in T2DM							
	Standard models				Multivariate interaction model^b^			
	Univariate		Multivariate^a^		Single term (main effects)		Interaction with HR	
	OR (95% CI)	*p*	OR (95% CI)	*p*	OR (95% CI)	*p*	OR (95% CI)	*p*
HR < 74.00								
BMI	1.24 (1.19–1.30)	<.001	1.17 (1.06–1.29)	.002	0.43 (0.18–1.10)	.076	1.01 (1.00–1.03)	.035
WC	1.08 (1.06–1.10)	<.001	1.07 (1.03–1.11)	.001	0.74 (0.54–1.02)	.065	1.01 (1.00–1.01)	.023
74.00 ≤ HR ≤ 88.00								
BMI	1.17 (1.13–1.20)	<.001	1.07 (1.01–1.14)	.021	1.07 (0.48–2.39)	.862	1.00 (0.99–1.01)	.999
WC	1.06 (1.05–1.07)	<.001	1.03 (1.01–1.06)	.003	1.01 (0.78–1.32)	.916	1.00 (0.99–1.01)	.890
HR > 88.00								
BMI	1.12 (1.08–1.17)	<.001	1.02 (0.94–1.11)	.643	1.14 (0.43–2.96)	.794	0.99 (0.99–1.01)	.825
WC	1.06 (1.04–1.07)	<.001	1.01 (0.98–1.05)	.361	1.21 (0.88–1.67)	.240	1.00 (0.99–1.00)	.271

^a^ Adjusted for: marriage + CHOL + educational level + AST + HC + HDL + SEX + SBP + T2DM_family + age + GGT.

^b^ As for multivariate standard model, adjusted for: marriage + CHOL + educational level + AST + HC + HDL + SEX+SBP + T2DM_family + age + GGT, but with the addition of interaction terms for BMI*HR or WC*HR.

Abbreviations: AST, aspartate aminotransferase; BMI, body mass index; CHOL, cholesterol; CI, confidence interval; GGT, γ‐glutamyltransferase; HC, hip circumference; HDL, high‐density lipoprotein; HR, heart rate; OR, odds ratio; SBP, systolic blood pressure; T2DM, type 2 diabetes mellitus; WC, waist circumference.

To test whether HR has a different relationship with the prevalence of T2DM, interaction terms for BMI × HR and WC × HR were entered in the regression models. The interaction between the major indicators of obesity (BMI and WC) and HR was statistically significant in patients with T2DM with HR < 74 beats/min. Interaction with HR for BMI was 1.01 (95% CI:1.00–1.03, *p* = .035), and Interaction with HR for WC was 1.01 (95% CI: 1.00–1.01, *p* = .023).

## 4 DISCUSSION

Our study discovered that HR was positively associated with prevalence of T2DM in the population with HR > 88 beats/minute. Exploring the association of BMI and WC with T2DM by different HR groups, we discovered that BMI and WC were strongly associated with higher risk of T2DM in those with HR ≤ 88 beats/minute. However, the interaction of HR with BMI and WC was significantly associated with the prevalence of T2DM only in those with HR < 74 beats/min.

The study indicated that HR > 80 beats/min was associated with increasing risk of T2DM. The results are similar to the studies of Hansen^24^ and Chunxiao Xu.^4^ The former study found that higher HR was associated with decreased insulin sensitivity, which may indicate that the increase of HR marked the subsequent deterioration of glucose metabolism; The latter study indicated that HR was independently associated with the prevalence rate of T2DM in rural China and it was a particularly evident among nonoverweight/obese participants. Due to the limitation of factors such as sample size, the HR grouping in previous studies was generally set to only HR = 80 beats/min, and the grouping of HR > 80 beats/min was not subdivided. Our study was intended to explore whether there was a more suitable risk threshold of HR for patients with T2DM in the range of HR > 80 beats/min. Consequently, we performed subgroup analysis according to results of restricted cubic spline and found that the prevalence of T2DM was significantly correlated with HR in participants with HR > 88 beats/min based on the results in the univariate and the multivariate regression analysis. Our study provides a more precise HR cutpoint for HR to assess the prevalence of T2DM and a basis for individualized assessment and targeted screening. Obesity as a state of excess energy promotes insulin resistance in tissues, and insulin resistance is the main cause of T2DM. In animal and human models of obesity, there were a lot of data on the relationship among insulin resistance, T2DM and obesity.^25^, ^26^, ^27^ Recently, some studies have shown that the relationship between obesity and T2DM was also affected by other factors, such as a study that found an interaction between autoimmune thyroid disease and obesity in patients with T2DM.^28^ A study of a Chinese rural population showed that low‐density lipoprotein receptor‐related protein 5 (LRP5) polymorphisms also interacted with obesity in T2DM, and LRP5 polymorphisms are associated with beta‐cell function and lipid metabolism.^29^ In addition, obesity and COVID‐19 interacted in the pathogenesis of diabetes.^30^ However, the evidence for the interaction of obesity and HR in the onset of T2DM was relatively lacking, with only one study on a rural population in Tongxiang, Zhejiang, China. Our study divides HR into three subgroups (HR < 74 beats/min, 74 ≤ HR ≤ 88 beats/minute, and HR > 88 beats/min) through restricted cubic spline models and explores the relationship and their interaction effect between BMI, WC, and T2DM under different HR groups. Our study indicated that BMI and WC were significantly associated with prevalence of T2DM when HR ≤ 88 beats/min. Moreover, only in the population with HR < 74 beats/min, the interaction of HR with BMI and WC was significantly associated with the prevalence of T2DM. Our results indicated that HR could be used as an independent assessment factor for prevalence of T2DM in the population with HR > 88 beats/min. In the population with HR < 74 beats/min, obesity‐related indicators can be used as an independent assessment factor for T2DM risk in this population.

The exact mechanisms and pathogenesis of HR and obesity on the prevalence rate of T2DM were not yet clear. HR was considered to be an important evaluation index of T2DM, and fast HR has a significant impact on mortality and prevalence of T2DM. The possible mechanisms include neuron injury and ischemia caused by hyperglycemia,^31^ autoimmunity,^32^ reduced protective effect of residual β cells,^33^, ^34^ and genetic factors.^35^ Obesity (BMI > 30 kg/m^2^) was a key component of type T2DM^36^ and was related to the development of insulin resistance by metabolic conditions,^37^ but the exact mechanism of obesity induced T2DM and insulin resistance still remains to be explained. Relevant studies^38^, ^39^ have shown that adipose tissue affects lipid and sugar metabolism through the release of nonesterified fatty acids (NEFAs) or other factors. The release of these molecules increases in obese people. Increasing NEFAs production leads to insulin insensitivity. The excessive production of NEFAs was found in obesity and T2DM and was related to IR in both cases.^40^ Soon after the increase of NEFA concentration in human plasma, insulin resistance began to develop,^41^ and insulin sensitivity was one of the important factors to calculate the fat distribution. There was evidence from longitudinal studies that sympathetic activation played a role in the development of obesity and that chronic sympathetic hyperactivity might contribute to the development of obesity by downregulating beta‐adrenergic receptor‐mediated thermogenic responses.^42^, ^43^ In addition, obesity was often accompanied by hyperinsulinemia, which could cause increased sympathetic tone and activate the renin‐angiotensin system, resulting in increased HR.^44^ These studies might prove that the interaction mechanism of HR and obesity on the occurrence of T2DM was related to sympathetic nerve.

According to the relevant information we have consulted, this was the most widely population‐based study on the independent and interactive effects of HR and obesity on the prevalence of T2DM at home and abroad. This study suggested the feasibility of HR and obesity measurement index as a reference index for T2DM screening and diagnosis and provided a reference for reevaluation of safe HR level of T2DM patients, offering the newly subdivided risk threshold of HR for patients with T2DM.

There were some limitations in our study. First, all studies adjusted for sex, age, marital status, DM family, educational level, CHOL, AST, HC, HDL, SBP, WC, and GGT. However, the residual confounding in the field investigation could not be completely eliminated, which might affect the study to a certain extent. For example, we could not exclude the effect of genetic differences in the population as there was a significant genetic susceptibility of T2DM. Second, due to the cross‐sectional design of our study, causal inference could not be obtained in these results. Further prospective studies are needed to clarify the precise relationship between HR, obesity, and T2DM risk. Third, the research population in this study was mainly female residents over 40 years old in the community, so the results might not represent other races and young people. To summarize, the results of this study need to be verified in other populations.

## 5 CONCLUSION

Our study suggested the feasibility of HR and obesity detection criteria (BMI and WC) as a reference index for T2DM screening and diagnosis and discovered their interaction in patients with T2DM with HR < 88beats/min. Meanwhile, it provided a reference for the newly subdivided risk threshold of HR for patients with T2DM, which might be HR = 88 beats/min.

## AUTHOR CONTRIBUTIONS

Conceived and designed the experiments: Meng Ren. Performed the experiments:Qingyu Chen, Tianxin Zhu, Hongxing Chen, Feng Li, Hongshi Wu, Juying Tang, Diaozhu Lin, Meng Ren. Analyzed the data: Lili You, Dan Liu, Kan Sun, and Xiaoyun Zhang. Wrote the manuscript: Tianxin Zhu and Meng Ren. All authors believe that the manuscript represents effective work, and the sixth final version has been reviewed and approved. The work has not been published before, and it is not considered to be published in whole or in part elsewhere.

## FUNDING INFORMATION

This work was supported by grants from the National Natural Science Foundation of China (U20A20352), the Guang Dong Clinical Research Center for Metabolic Diseases (2020B1111170009), and the Key‐Area Research and Development Program of Guangdong Province (2019B020230001). The funders were not involved in research design, data collection and analysis, preparation of manuscripts or decision to publish.

## DISCLOSURE

All of the authors declare that they have no relevant conflicts of interests.
